# Creation of a pediatric mature B-cell non-Hodgkin lymphoma cohort within the Pediatric Health Information System Database

**DOI:** 10.1371/journal.pone.0186960

**Published:** 2017-10-23

**Authors:** Rebecca Citrin, Joseph P. Horowitz, Anne F. Reilly, Yimei Li, Yuan-Shung Huang, Kelly D. Getz, Alix E. Seif, Brian T. Fisher, Richard Aplenc

**Affiliations:** 1 Division of Oncology, The Children’s Hospital of Philadelphia, Philadelphia, Pennsylvania, United States of America; 2 Center for Clinical Epidemiology and Biostatistics, Perelman School of Medicine at the University of Pennsylvania, Philadelphia, Pennsylvania, United States of America; 3 Drexel University College of Medicine, Philadelphia, Pennsylvania, United States of America; 4 Department of Pediatrics, Perelman School of Medicine at the University of Pennsylvania, Philadelphia, Pennsylvania, United States of America; 5 Center for Pediatric Clinical Effectiveness, The Children’s Hospital of Philadelphia, Philadelphia, Pennsylvania, United States of America; 6 Division of Infectious Diseases, The Children’s Hospital of Philadelphia, Philadelphia, Pennsylvania, United States of America; Universidad de Navarra, SPAIN

## Abstract

Mature B-cell non-Hodgkin lymphoma (B-NHL) constitutes a collection of relatively rare pediatric malignancies. In order to utilize administrative data to perform large-scale epidemiologic studies within this population, a two-step process was used to assemble a 12-year cohort of B-NHL patients treated between 2004 and 2015 within the Pediatric Health Information System database. Patients were identified by ICD-9 codes, and their chemotherapy data were then manually reviewed against standard B-NHL treatment regimens. A total of 1,409 patients were eligible for cohort inclusion. This process was validated at a single center, utilizing both an institutional tumor registry and medical record review as the gold standards. The validation demonstrated appropriate sensitivity (91.5%) and positive predictive value (95.1%) to allow for the future use of this cohort for epidemiologic and comparative effectiveness research.

## Introduction

Pediatric mature B cell non-Hodgkin lymphomas (B-NHL) represent 4% of all pediatric malignancies, with an annual incidence of approximately 450 cases in the United States [1]. The relative rarity of these malignancies makes them difficult to study outside of large cooperative group trials. Administrative databases are an alternative data source within which to perform large-scale epidemiologic and comparative effectiveness studies. However, the use of administrative databases to study B-NHL has been hampered by well-described limitations of ICD-9 codes in identifying newly diagnosed pediatric cancer patients [2–4]. To address this limitation, we developed a multistep process incorporating ICD-9 codes and manual review of chemotherapy billing data to assemble and validate a cohort of pediatric patients with *de novo* B-NHL using the Pediatric Health Information System (PHIS) database.

## Materials and methods

### Data source

PHIS is a comprehensive administrative database containing inpatient and emergency department data from 48 freestanding children’s hospitals throughout the United States. The available data is comprised of patient-level information including demographics, dates of admission, discharge disposition, and all ICD-9 diagnosis and procedure codes linked to a given admission. It also contains billed resource utilization data, which incorporates pharmaceuticals, laboratory tests, imaging studies, procedures, and the dates that these bills were generated. Patients are assigned a unique identifier within this database, allowing for identification across multiple inpatient admissions within the same institution. This study was approved by the Children’s Hospital of Philadelphia’s (CHOP) Institutional Review Board and was granted a waiver of informed consent.

### Cohort assembly

First, all admissions to one of the 48 contributing PHIS sites between January 1, 2004 and September 30, 2015 were screened for the presence of an ICD-9 diagnosis code consistent with possible B-NHL. All relevant ICD-9 codes utilized are listed in Table 1, along with the analogous ICD-10 codes for future database queries. Patients were excluded if they lacked billing data for all thirteen pre-specified chemotherapeutic agents used in standard American B-NHL treatment regimens.

**Table 1:** List of ICD-9 and ICD-10 diagnosis codes for B-NHL.

**ICD-9 code**	**Description**
200.0x	Reticulosarcoma
200.2x	Burkitt’s leukemia or lymphoma
200.5x	Primary CNS lymphoma
200.7x	Large cell lymphoma
200.8x	Other named variants of reticulosarcoma and lymphosarcoma
202.8x	Other malignant lymphomas
202.9x	Other and unspecified malignant neoplasms of lymphoid and histiocytic tissue
**ICD-10 code**	**Description**
C83.3x	Diffuse large B cell lymphoma
C83.7x	Burkitt’s lymphoma
C83.8x	Other non-follicular lymphoma
C83.9x	Non-follicular (diffuse) lymphoma
C85.1x	Unspecified B cell lymphoma
C85.2x	Mediastinal (thymic) B cell lymphoma
C85.8x	Other specified types of non-Hodgkin lymphoma
C85.9x	Non-Hodgkin lymphoma, unspecified
C96.9	Malignant neoplasm of lymphoid, hematopoietic, and related tissue, unspecified
C96.Z	Other specified malignant neoplasms of lymphoid, hematopoietic, and related tissue

Next, chemotherapy data were manually reviewed for at least 45 days from the first hospital admission associated with the malignant diagnosis code. Patients were included in the final cohort if they had at least two courses of chemotherapy consistent with established B-NHL regimens. Specifically, the majority of these regimens included one COPADM (cyclophosphamide, vincristine, doxorubicin, methotrexate, and either prednisone, prednisolone, or methylprednisolone) or RCOPADM (COPADM + rituximab), with or without a preceding COP cytoreduction cycle (cyclophosphamide, vincristine, and either prednisone, prednisolone, or methylprednisolone). Additionally, patients who received two COPAD courses (COPADM without methotrexate), at least two R-EPOCH cycles with or without a preceding COP reduction cycle (rituximab, etoposide, vincristine, cyclophosphamide, doxorubicin, and either prednisone, prednisolone, or methylprednisolone), or consecutive regimens per the non-Hodgkin Lymphoma Berlin-Frankfurt-Munster (BFM) 95 study (course 1: methotrexate, cytarabine, etoposide, ifosfamide, dexamethasone, +/- vincristine; course 2: cyclophosphamide, doxorubicin, methotrexate, dexamethasone, +/- vincristine) were eligible for cohort inclusion. These regimens were selected based upon their use in the following cooperative group trials during or preceding the study period: Children’s Cancer Group 5961, Children’s Oncology Group ANHL01P1, Children’s Oncology Group ANHL1131, and Berlin-Frankfurt-Munster NHL 95 trial [5–8]. This cohort generation process allowed for subtle deviations in chemotherapy patterns, including any number of additional days of steroid exposure, adjustments in intra-cycle chemotherapy schedules of up to two days, and the absence of up to one chemotherapy class per cycle.

Additionally, patients were eligible for cohort inclusion if they received at least one COP reduction cycle (without the requirement to receive at least two cycles) with no subsequent inpatient encounters in at least the following 45 days. This condition was stipulated in order to capture patients who may not live long enough to meet the full inclusion criteria detailed above. Lastly, patients who received cycles of B-NHL directed therapies that typically appear later in cooperative group protocols underwent additional review, with entries into the PHIS database reviewed for 60 days prior to the initial admission identified by ICD-9 codes. These patients were included in the cohort if the subsequent review identified earlier chemotherapy regimens meeting inclusion criteria. Patients who did not receive chemotherapy as detailed above were excluded from the primary cohort.

Two authors (R.C. and J.P.H) conducted the primary chemotherapy review. To evaluate inter-rater reliability, 10% of the sample was re-reviewed by both evaluators and a κ-statistic was calculated as a measure of inter-rater agreement with regard to cohort inclusion status.

### Cohort validation

Cohort validation utilized the medical records of patients with *de novo* B-NHL treated at CHOP as well as an institutional tumor registry maintained by CHOP. In order to calculate the positive predictive value (PPV) of inclusion in the cohort, members of the study team reviewed pathology reports, attending oncologists’ admission notes and discharge summaries, and medication administration records of all CHOP treated patients identified in the initial PHIS database ICD-9 code query. The sensitivity was calculated by comparing the patients identified in PHIS to an institutional CHOP registry of patients with B-NHL.

Finally, in order to ensure that this was a representative cohort, demographic characteristics of patients in the final cohort were extracted from PHIS (including age, gender, and race), and were compared with those reported by the National Cancer Institutes’ SEER Program from 2004–2013 using Pearson’s χ^2^ test [9]. All analyses were performed using Microsoft Excel, SAS version 9.3 (Cary, NC), and STATA statistical software version 14.0 (College Station, TX)

## Results

**Fig 1:**
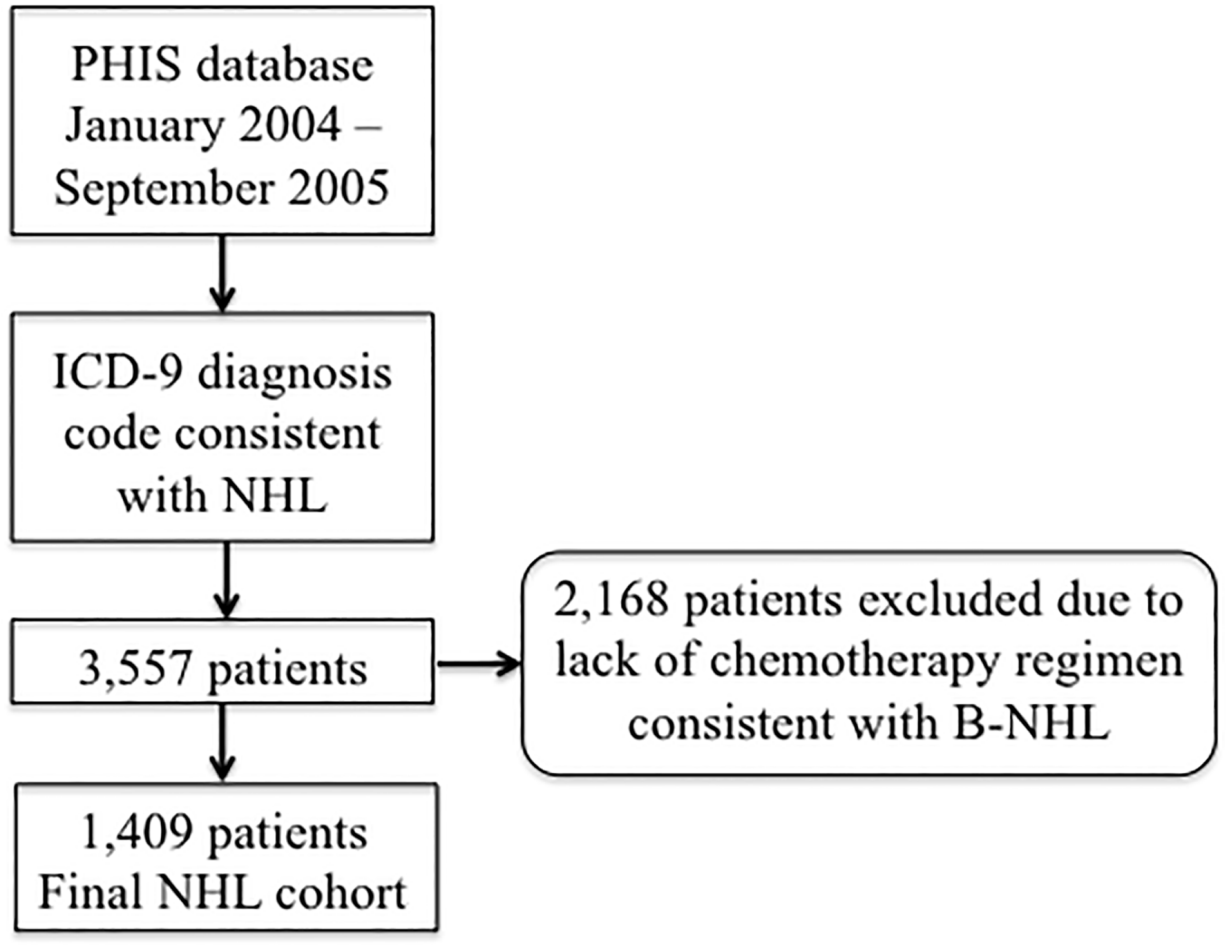
Flow chart depicting the cohort assembly process.

A total of 3,557 unique patients with an ICD-9 code for B-NHL were identified in PHIS within the study period. All patients underwent manual chemotherapy review, with 1,409 patients remaining eligible for cohort inclusion. This process is depicted in Fig 1 and Table 2. The 10% of the cohort reviewed by both reviewers had inter-rater concordance of 99.4%.

**Table 2:** Validation results from each step of the generation process of a pediatric mature B cell non-Hodgkin lymphoma cohort.

	True positives	False positives	False negatives	Sensitivity (95% CI)	Positive predictive value (95% CI)
Step 1: Identification via ICD-9 codes (200.0, 200.2, 200.5, 200.7, 200.8, 202.8, 202.9)	103	70	5	95.4% (89.5–98.5%)	59.5% (51.8–66.9%)
Step 2: Manual chemotherapy review*	97	5	9	91.5% (84.5–96.0%)	95.1% (88.9% - 98.4%)

*The validation cohort for sensitivity and PPV calculations included 178 patients aged 0–21 treated at CHOP during the study period.

This two-stage process of cohort generation was validated within the 178 (5%) patients treated at CHOP. As shown in Table 2, using ICD-9 codes alone resulted in a sensitivity of 95.4% with a PPV of 59.5%. Manual chemotherapy review increased the PPV of the primary cohort to 95.1%, with a corresponding decrease in sensitivity to 91.5%. Prior to manual chemotherapy review, the majority (85.7%) of the false positive cases were patients with alternate diagnoses, largely T-cell lymphomas and Hodgkin lymphoma. Smaller subsets consisted of patients with relapsed disease (1.4%), patients who began their treatment at a non-PHIS institution and were transferred for second opinions or continuation of therapy (10.0%), and patients who had no evidence of malignancy (2.9%). Following manual chemotherapy review, the five false positive cases included one patient with relapsed disease and four patients with alternate diagnoses treated on B-NHL regimens. The nine false negatives were comprised of five patients who were not identified in the PHIS database ICD-9 code query and four patients who received altered chemotherapy regimens due to comorbidities. There were statistically significant differences in demographic characteristics of patients identified in PHIS as compared to those from the National Cancer Institute’s SEER Registry for years 2004–2013 (Table 3).

**Table 3:** Demographic characteristics of patients in PHIS-assembled cohort compared to the National Cancer Institute’s SEER program.

	PHIS-assembled (n = 1,395*)		SEER 2004–2013 (n = 1,477)		*P*-value
	Number	%	Number	%	
**Age category**					
<1	2	0.14	4	0.27	<0.001
≥1 and <5	182	13.1	170	11.51	
≥5 and <10	374	26.8	301	20.38	
≥10 and <15	434	31.1	402	27.22	
≥15 and <20	403	28.9	600	40.62	
**Gender**					
Male	1,058	75.8	1007	68.18	<0.001
Female	337	24.2	470	31.82	
**Race**					
White	951	68.2	1131	76.57	0.03
Black	131	9.4	203	13.74	
Other**	294	21.1	130	8.8	
Unknown**	19	1.4	13	0.9	

*14 subjects aged 20–21 in the PHIS-assembled cohort are excluded from all comparisons, as the NCI SEER program does not include this specific age range.

**Other/unknown categories were excluded from this comparison

## Discussion

This study outlines the derivation and validation process of assembling a cohort of children with B-NHL within a large administrative database. Our group has previously reported that using ICD-9 diagnosis codes alone confers a poor positive predictive value for accurate patient identification in pediatric malignancies [2–4]. Our low initial PPV based on ICD-9 diagnosis codes alone further underscores the importance of combining chemotherapy review with ICD-9 diagnosis codes in order to accurately identify patients with B-NHL.

Aside from the many potential advantages of this cohort, there are some important limitations. First, demographic parameters within our cohort demonstrate statistically significant differences from those reported by the SEER program across all categories, thus threatening the generalizability of this cohort. One potential explanation for this discrepancy is that older teenagers and young adults may be treated at either pediatric or adult institutions, only the former of which is captured by PHIS. This hypothesis is supported by secondary comparisons of our cohort to SEER data, with the age range restricted to patients <15 years old. The supplemental analysis negated this difference for both age and race categories, with a persistent statistically significant difference noted in the proportions of male and female patients (77.5% male patients in our cohort compared to 71.7% in SEER data; p = 0.004). This finding suggests that the generalizability of our cohort is likely to be preserved for patients treated at children’s hospitals, but may not extend to those treated at adult institutions.

Second, we *a priori* excluded certain mature B cell non-Hodgkin lymphomas, including follicular lymphoma, mantle zone lymphoma, and other similar malignancies. These malignancies were excluded due to their rarity among pediatric populations as well as their treatment heterogeneity. While this exclusion also has the potential to limit the generalizability of our cohort, we expect this effect to be minimal due to the infrequency of these conditions in children.

Third, the nature of our two-step patient identification process creates the potential to erroneously exclude patients from the cohort (false negatives). Specifically, this process relies on accurate assignment of ICD-9 diagnosis codes, as well as comprehensive chemotherapy billing and reporting. It is possible that institutions have different processes for assignment of ICD-9 diagnosis codes as well as different treatment and pharmacy billing practices, which could result in misclassification. However, our validation cohort showed a robust sensitivity, suggesting that the risk of falsely excluding patients was likely small. Furthermore, this approach yielded a high PPV, indicating that the patients included in the final cohort truly had B-NHL. Notably, the validation was only conducted at a single center and thus there is the potential for altered sensitivity and PPV calculations if the validation were conducted at multiple sites. However, the demographic similarities of our cohort to SEER data suggest that the risk of false negatives is likely small.

Lastly, the use of ICD-9 codes for initial patient identification may make this process difficult to repeat in the era of ICD-10 coding. As detailed in Table 1, all ICD-9 codes used in this process have comparable ICD-10 codes, most with higher level of detail. Given these similarities, we anticipate that utilization of ICD-10 codes will generate a similarly representative patient cohort.

Despite these limitations, this cohort represents a unique opportunity to perform large-scale comparative effectiveness and epidemiologic research studies on an uncommon collection of pediatric malignancies.
